# Case report: Venetoclax therapy in a boy with acute myeloid leukemia in Shwachman Diamond syndrome

**DOI:** 10.3389/fped.2022.1059569

**Published:** 2023-01-09

**Authors:** Samuele Naviglio, Antonio Giacomo Grasso, Chiara Iacono, Giada Zanella, Valentina Kiren, Nagua Giurici, Federico Verzegnassi, Natalia Maximova, Marco Rabusin

**Affiliations:** ^1^ Pediatric Oncology and Hematology Department, Institute for Maternal and Child Health IRCCS “Burlo Garofolo”, Trieste, Italy; ^2^ Department of Medicine, Surgery and Health Sciences, University of Trieste, Trieste, Italy

**Keywords:** Shwachman Diamond syndrome, venetoclax, acute myeloid leukemia, myelodysplastic syndromes, pediatric

## Abstract

Shwachman-Diamond syndrome (SDS) is a rare bone marrow failure syndrome characterized by exocrine pancreatic insufficiency, bone abnormalities, progressive cytopenia, and predispositions to myelodysplastic syndrome (MDS) and acute myeloid leukemia (AML). AML, in these patients, is associated with a poor prognosis and with an increased risk of organ toxicity and infectious complications from chemotherapy and hematopoietic stem cell transplantation (HSCT), thus leading to high rates of treatment-related morbidity and mortality. The BCL-2 inhibitor venetoclax has revolutionized the treatment of AML in elderly adults, especially for treatment-naive elderly patients who are ineligible for intensive chemotherapy. There is limited evidence on the use of venetoclax in pediatric patients with SDS-related MDS or AML. Here, we report a case of a 14-year-old boy with SDS with AML arising from MDS. The patient was treated with two cycles of conventional chemotherapy with fludarabine and cytarabine with an initial good response but immediate relapse and substantial toxicity. Treatment with venetoclax and azacitidine was started, with a substantial reduction of leukemic burden (good response on peripheral leukemic infiltration and partial response in the bone marrow after one course). However, it was followed by multiple infectious complications and worsening of the general condition not allowing treatment to be continued, and the patient eventually died from multiorgan failure. With the limitations of observation of a single patient, our experience suggests that venetoclax/azacitidine combination therapy may represent a therapeutic possibility for patients with SDS and AML, even though it may be associated with significant toxicity.

## Introduction

Pediatric myelodysplastic syndromes (MDS) are clonal affections of hematopoiesis leading to refractory cytopenia, abnormal maturation of hematopoietic precursors, and risk of evolution to acute myeloid leukemia (AML) (1). Shwachman Diamond syndrome (SDS) is a rare autosomal recessive disease characterized by abnormal hematopoiesis, pancreatic insufficiency, and bone alterations (2, 3). Almost all children with SDS have moderate-to-severe neutropenia and about half of them will also experience some degree of anemia or thrombocytopenia due to inefficient bone marrow production (4). A real MDS may eventually ensue, affecting 19%–25% of patients at 20 years of age and up to 36%–40% at 30 years of age, with some patients evolving to AML (5). How MDS appears and subsequently progresses to AML is still not clear. The main hypotheses implicate an unbalance between pro-apoptotic and anti-apoptotic signals (such as BCL2) in the stressed bone marrow (6, 7). Moreover the SBDS protein may be particularly important in stabilizing the mitotic spindle (8). The prognosis of SDS patients with MDS/AML is dismal, and their management is challenging. Chemotherapy alone is not a feasible approach due to the difficulty of regenerating normal hematopoiesis, the high incidence of relapse or new clonal evolution, and the increased risk of toxicity (mostly infections) associated with standard AML therapeutic protocols (9, 10). As a result, the only curative option remains hematopoietic stem cell transplantation (HSCT). SDS patients are at an increased risk of transplant-related complications and mortality, including cardiac failure during conditioning with cyclophosphamide and Graft versus Host Disease (11, 12). The role of pre-transplant chemotherapy as a mean to reduce disease load is still unclear. In fact, it is important to find a balance between the need to reduce the leukemia burden and avoiding excess toxicity. In the last years, hypomethylating agents such as azacitidine and decitabine have shown promising results in patients with MDS, particularly when clinical conditions do not allow the use of more conventional chemotherapy approaches (13, 14). Recently, these compounds have been associated with new agents like venetoclax, a selective inhibitor of BCL2, an anti-apoptotic protein that is over-expressed in some AML cells that are BCL2-dependent for survival (15). Although venetoclax alone has little direct activity on AML cells, synergic treatment with a hypomethylating agent can sensitize blast cells to the drug's pro-apoptotic activity (16). Recent data from treatment-naive, elderly AML patients with combined chemotherapy consisting of venetoclax and hypomethylating agents have shown promising outcomes (17). Little data is available on pediatric patients, and their use in patients with SDS is anecdotal. Here we describe the case of a boy affected by SDS-associated MDS/AML who was treated with conventional chemotherapy and then with venetoclax/azacitidine as a bridge therapy to HSCT.

## Case presentation

**Figure 1:**
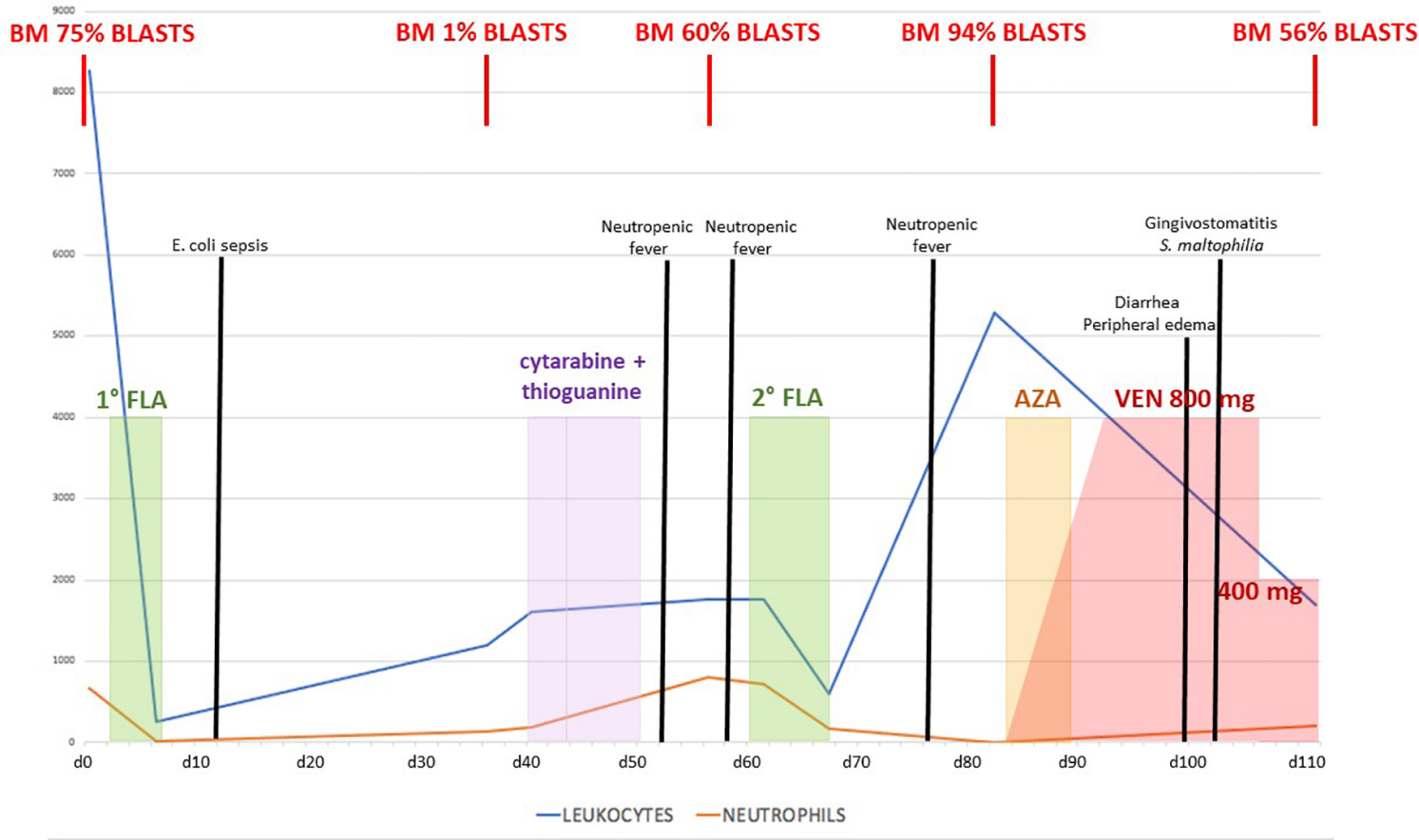
Graph presentation of chemotherapy treatment with blast count trend and clinical course complications.

We report the case of a 14-year-old boy affected by SDS that had been diagnosed in the first year of life with neutropenia, failure to thrive, and recurrent respiratory infections. In the following years, he had always been well, with moderate-to-severe neutropenia (400–600/mmc) but without severe infections. Yearly hematologic controls were performed, including regular bone marrow aspirates, with no evidence of myelodysplastic progression. When the patient was 16 years and 7 months old, he was admitted for persistent fever without other symptoms. Blood tests showed trilinear cytopenia: therefore, a bone marrow aspirate was performed, which showed a hypercellular marrow with the prevalence of erythroid precursors (50%), often with dysplastic signs, suggesting MDS. A bone marrow biopsy confirmed the presence of a hypercellular MDS with the prevalence of an immature erythroid population and an increase of megakaryocytes, which appeared dysplastic; flow cytometry analysis showed 8% of myeloid blasts (MDS with excess blasts—MDS EB1 according to WHO 2017). The recommendation to proceed to HSCT was formalized, and since he did not have compatible HLA siblings, a search for a matched unrelated donor (MUD) was started without starting any MDS-directed therapy. One month after the diagnosis, while waiting for the HSCT, he developed splenomegaly (2 cm below the left costal margin) and peripheral monocytosis. Repeated bone marrow aspirate showed progression to AML (bone marrow flow cytometry showed 75% blasts CD34+/CD7+/CD45+/CD33+/CD11b dim/CD117+). Cytogenetic and molecular analysis (including RNA and DNA analysis for the main translocations associated with AML) showed no abnormalities. Treatment with Fludarabine—Cytarabine (FLA) was started (fludarabine 30 mg/m^2^/day and cytarabine 2,000mg/m^2^/day for 5 days), aiming to achieve leukemic burden reduction in view of HSCT. Treatment was well tolerated overall with no major organ toxicity, yet it was followed by an *Escherichia coli* sepsis requiring hospital admission (see Figure 1 for the overall clinical course). Bone marrow re-evaluation showed morphologic remission (blasts 1%, confirmed by flow cytometry), with the persistence of abnormal hematopoiesis, compatible with pre-existent MDS. Maintenance therapy with low-dose i.v. cytarabine plus oral thioguanine was started, which was nevertheless discontinued due to worsening thrombocytopenia requiring platelet transfusions. Second hospital admission was required due to mucositis and febrile neutropenia, which responded to antibiotic therapy. Nevertheless, while arrangements for the MUD HSCT were being organized, AML relapsed (60% blasts in the bone marrow). Therefore a second FLA cycle was administered. This was again followed by neutropenic fever requiring hospitalization. After an initial improvement with antibiotic therapy, he developed persistent fever associated with marked hepatosplenomegaly and ascites [grade 2 according to Common Terminology Criteria for Adverse Events (CTCAE) v. 5.0]. Peripheral blood flow cytometry showed leukocytosis with 93% blasts, and bone marrow aspirates confirmed persistent massive leukemic infiltration (94% blasts). In consideration of the occurrence of severe toxicity from conventional chemotherapies, a second-line chemotherapy regimen was considered too dangerous. Therefore combination therapy with azacitidine (125 mg/day for 7 days) and venetoclax for 28 days was started, aiming to achieve disease control before proceeding to transplant. Venetoclax was started at a dose of 100 mg/day and gradually increased by 100 mg/day up to a dose of 800 mg/day. During the first 10 days of therapy, a gradual decrease of blasts in the peripheral blood was seen, with a marked decrease in hepatosplenomegaly. Therapy was overall well tolerated but was associated with diarrhea (grade 2 according to CTCAE v 5.0) and peripheral edema (grade 2 according to CTCAE v 5.0), a known side effect of venetoclax, as well as with an urticarial rash that resolved upon azacitidine discontinuation. Bone marrow examination performed at the end of the therapeutic course showed partial response on leukemic infiltration: the bone marrow appeared markedly hypocellular, thereby showing a definite response on AML burden in absolute terms (as compared to pre-treatment evaluation), even though leukemic blasts were still 52% by flow cytometry. Unfortunately, the patient developed a series of infectious complications following the end of the course, including severe *Stenotrophomonas maltophilia* gingivostomatitis (grade 3 according to CTCAE v 5.0), followed by two episodes of sepsis by *Staphylococcus epidermidis* and *Escherichia coli*, respectively. An increase of hepatosplenomegaly was also observed, likely due to AML progression, together with marked fluid overload only partially responding to diuretic therapy, and he was considered not eligible for HSCT or to continue therapy with a combination of azacitidine and venetoclax. Palliative therapy with azacitidine alone was started, with no response, and he eventually died from multiorgan failure.

## Discussion

SDS is a rare and complex disease, and management of hematological manifestations can be challenging due to the absence of dedicated protocols and the intrinsic frailty of these patients. Often these patients undergo annual surveillance for MDS/AML with bone marrow, although the utility of this approach is controversial (18). When MDS occurs, nevertheless, the outcome is often poor. In the largest series of SDS patients with advanced MDS-AML, overall survival ranged from 10% for AML to 40% for MDS. In particular, mortality was due both to relapsed disease and to chemotherapy and transplantation-related toxicity, with almost half of patients dying before reaching HSCT (12–20). There is no consensus on the role of pre-HSCT cytoreductive chemotherapy, and data is sparse. When AML was diagnosed, we initially decided to use the FLA protocol due to the rapid progression of leukemia. Notably, AML in our patient showed a very good response to the first FLA course, achieving complete morphological and cytofluorimetric remission. Unfortunately, as may be expected in a disease prone to accumulate new mutations, AML relapsed and became refractory to FLA chemotherapy. Although we did not perform a second cytogenetic analysis at relapse, it is possible that AML cells had acquired a complex karyotype, as described in refractory disease (21). Given worsening toxicity from chemotherapy courses, we chose to try a combination of venetoclax and azacitidine, based on adult experience and small pediatric case series (22–24). There is little data on experience with venetoclax for myeloid malignancies in children. The largest case series, reported by Winters et al., includes eight patients treated with venetoclax and azacitidine (two for MDS and six for AML), with morphologic response in six patients, including both patients with MDS and four AML patients. AML patients who responded did achieve negative minimal residual disease. Three patients eventually underwent HSCT. Therapy was well tolerated in all patients, and the most common adverse events were hematologic and gastrointestinal. One of the two patients with MDS was a 7-year-old girl with SDS who had developed MDS with excess blasts and monosomy 7. She was treated with venetoclax (at 800 mg/day-equivalent dose adjusted on body weight) and azacitidine 75 mg/m^2^) with good response and reduction of bone marrow blast percentage to less than 1%. Notably, she underwent cord blood HSCT with primary graft failure and then proceeded to haploidentical HSCT with minimal toxicity after a conditioning regimen consisting of fludarabine, total body irradiation, anti-thymocyte globulin, and cyclophosphamide. In our patient, after the first 2 weeks of combination therapy, we witnessed a complete response in peripheral blast count and a marked reduction of hepatosplenomegaly. Bone marrow re-evaluation after the first month of therapy showed a partial response in relative terms (reduction of blasts from 92% to 52%) but with a marked reduction of AML burden, as the marrow appeared frankly hypocellular. Furthermore, although the response was not complete, the boy had received only one cycle of therapy: experience in adult patients treated with venetoclax/azacitidine for untreated AML showed a complete response after only one cycle in 40% of patients, reaching 66% after the second cycle with a median number of completed 28 days cycles of 7 (21, 25). It is possible to speculate therefore that another cycle could have further reduced the blast count prior to the HSCT. Notably, however, even though venetoclax/azacitidine has been shown to be well-tolerated even in frail patients, our patient developed several infectious complications and significant fluid retention, which are known toxicities of azacitidine/venetoclax therapy, eventually not allowing to proceed with HSCT. Future trials are needed to try to optimize venetoclax dosing strategies in frail patients, as reduced dosing might have been associated with better tolerance.

Overall, his clinical course was characterized by extreme instability and evanescence of response to therapies, with rapid relapse even after effective treatments. This was likely due to the absence of normal hematopoiesis in MDS arising in the context of the underlying SDS. In hindsight, considering the instability of the hematologic response in these patients, proceeding to HSCT at first disease remission (i.e., after the first FLA) could possibly have represented the best chance for this patient, albeit with all the limits and caveats associated with the need of using a haploidentical donor. Although haploidentical HSCT has been considered inferior to MUD for a long time, recent advances have led to similar outcomes, making this approach feasible for a wide type of diseases including AML patients (26). Specific data for SDS, nevertheless, are scarce. Cyclophosphamide, which is used for T-cell depletion *in vivo*, has been used in an adult patient with SDS and AML, suggesting that this method may be applicable also for patients with SDS, yet with some risk of cardiotoxicity (27). Another approach could be alpha/beta T-cell depletion, but experience in literature is limited for SDS, and cellular manipulation *ex vivo* was not available in our center.

## Conclusion

Patients with SDS that develop MDS and AML are prone to high morbidity and mortality due to severe treatment-related toxicity and relapsed/refractory disease. With the limitations of observation of a single patient, our experience suggests that AML in SDS may respond to venetoclax/azacitidine combination therapy, which could therefore represent an option as a bridge to HSCT. Further studies are necessary to define the best treatment regimens and hopefully shared treatment recommendations for MDS/AML in these patients.

## Data Availability

The original contributions presented in the study are included in the article/Supplementary Material, further inquiries can be directed to the corresponding author.
